# Superior Mesenteric Artery Syndrome Managed with Laparoscopic Duodenojejunostomy

**DOI:** 10.1155/2022/4607440

**Published:** 2022-08-03

**Authors:** Ahmed Sabry, Ramy Shaalan, Carl Kahlin, Ahmed Elhoofy

**Affiliations:** ^1^Department of General Surgery, Faculty of Medicine, Ain Shams University, Cairo, Egypt; ^2^Surgical Department, Ninewells Hospital, NHS Tayside, Dundee, UK; ^3^Vascular Surgery Department, Ninewells Hospital, NHS Tayside, Dundee, UK

## Abstract

**Background:**

Superior mesenteric artery (SMA) syndrome is a rare disorder that may be managed surgically if conservative management fails. Different surgical techniques have been described, division of the ligament of Treitz, gastrojejunostomy, and duodenojejunostomy. The aim of this case series is to show that laparoscopic duodenojejunostomy is a safe and technically feasible management for superior mesenteric artery syndrome.

**Methods:**

In this case series, we retrospectively identified all patients who underwent laparoscopic duodenojejunostomy for SMA syndrome in our tertiary university center between December 2016 and July 2019. Data collected included demographics, presenting symptoms, comorbidities, pre and postoperative body mass index (BMI), operative approach, operative blood loss, operative duration, clinical and radiological results, in hospital/30-day complications, mortality, and postoperative follow-up outcomes.

**Results:**

We identified eleven patients, 10 females and 1 male, with a median age 23 years (range 17–43 years). All patients had refractory symptoms after a minimum of two months of conservative management and subsequently underwent laparoscopic duodenojejunostomy. There were no intraoperative complications and no in-hospital or 30-day postoperative mortality or complications were identified. Follow-up data showed complete resolution in 73% of patients (*n* = 8) and only one patient with no improvement postoperatively. Results also showed a median BMI increase of 2 kg/m^2^ (range 1–9 kg/m^2^) at a median follow-up of 16 months (range 4–48 months).

**Conclusion:**

Laparoscopic duodenojejunostomy is a safe treatment option for SMA syndrome and should be considered when patients do not respond to conservative management.

## 1. Introduction

Superior mesenteric artery syndrome (SMAS) is a rare condition where the third portion of the duodenum is compressed between the vertebral column and aorta posteriorly and the superior mesenteric artery (SMA) anteriorly as it originates from the aorta at an acute angle.

The syndrome is originally described by Rokitansky in 1861, but Wilkie was the first to publish a big series of 75 patients in 1927, hence the eponymous name “Wilkie's Syndrome” [1].

There is an established association between rapid weight loss and the development of SMAS. This is likely due to loss of the mesenteric fat pad separating the SMA from the aorta causing the increased acuity of the angle between the two structures and the subsequent duodenal compression [2]. Other predisposing conditions are spinal surgeries, psychiatric illness, or other wasting conditions such as malabsorption, burns, cancer, paraplegia, and tuberculosis [3–5].

Patients most commonly present with anorexia, early satiety, postprandial abdominal pain, and fullness and bilious vomiting [6].

**Figure 1:**
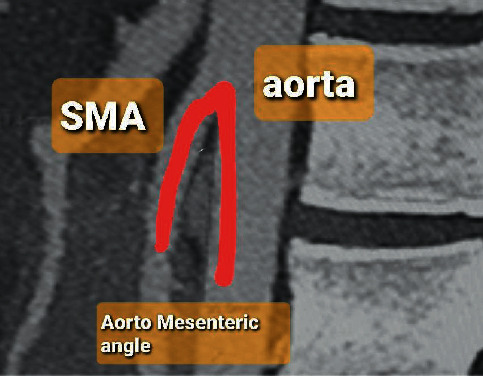
CT scan, sagittal view, showing an aortomesenteric angle of 13°.

**Figure 2:**
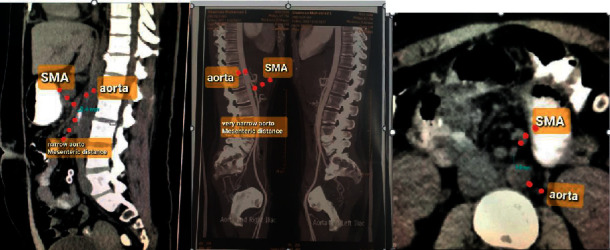
CT sagittal and coronal views showing narrow aortomesenteric distance.

**Figure 3:**
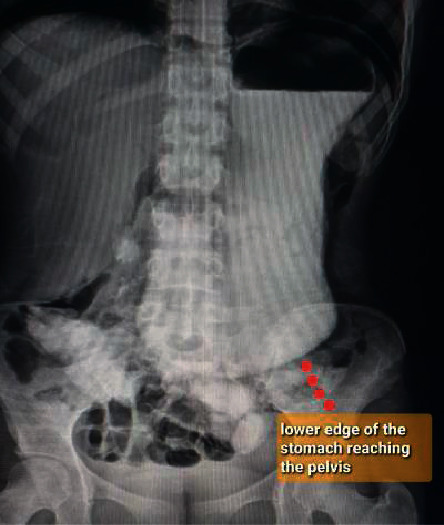
A contrast meal showing hugely dilated stomach extending down to the pelvis.

Diagnosis of SMAS is usually delayed due to lack of awareness of the condition which prolongs patients' suffering. The mainstay of the diagnosis is radiological via computed tomography (CT) scan with mesenteric angiography to confirm the aortomesenteric angle ≤25° and aortomesenteric distance ≤8 mm where the SMA crosses the duodenum [3]. Other investigations include upper gastrointestinal endoscopy and contrast meal (Figures1–3).

The first line of management of SMAS is conservative, mainly aiming to regain weight to restore the mesenteric fat pad and subsequently increase the aortomesenteric angle [7]. Reports suggested success rate as high as 71.3% in a big series of 80 patients; however, there was risk of recurrence [3].

Surgical management is recommended after failure of conservative management. Multiple procedures were described including release of the ligament of Treitz “Strong's procedure,” gastrojejunostomy, and duodenojejunostomy. The latter is currently the most widely accepted procedure. With the growing skills and advances in the bariatric field, the laparoscopic technique is becoming the gold standard which is both feasible and safe [2], [8].

Due to the scarcity of cases, most of the published evidence is descriptive outcome studies with small number of cases and short follow-up. In addition, some of the population cohorts with special characteristics and dietary habits, like middle east and African regions, are not represented. Laparoscopic duodenojejunostomy was described in the literature with multiple variations including the patient set up and anastomosis technique. There was also no standardization of the surgeon's training and specialty for such a rare operation. Our assumption is that the best outcome is likely to be in the fully trained bariatric hands given the familiarity with anatomy and intracorporeal anastomosis.

To our knowledge, this study is the largest case series published describing the surgical management of SMAS with laparoscopic duodenojejunostomy in a Middle Eastern and African cohort. All cases were performed by fully trained bariatric surgeons following the standardized technique.

## 2. Methods

We identified all patients who underwent laparoscopic duodenojejunostomy as definitive management for SMA syndrome following failure of conservative management in our tertiary university centre's surgical database between December 2016 and July 2019. An analysis of retrospectively collected data was performed. Median follow-up was 18 months (12–30 months).

### 2.1. Preoperative Work-Up

For all eleven cases, diagnosis was confirmed radiologically. Using arterial phase reconstruction, CT scan criteria for diagnosis included aortomesenteric angle <25° (Figures 1 and 2). Two of the patients who had borderline aortomesenteric angles underwent contrast meal which supported the diagnosis of SMAS (Figure 3).

During work up, all patients underwent upper gastrointestinal endoscopy to exclude other pathologies. Psychiatric consultation was also arranged for all patients to rule out eating disorders.

Prior to consideration for surgery, all patients underwent a period of at least two months of conservative management under the care of the local gastroenterology team. However, the eleven cases in our study did not have significant improvement with conservative management. The eleven patients were evaluated by our multidisciplinary team (MDT) of consultants including gastrointestinal surgery, vascular surgery, gastroenterology, and radiology. Consensus was achieved from the MDT before proceeding to surgery.

### 2.2. Data Collection

Patient baseline demographics including age, sex, body mass index (BMI), duration of symptoms, and comorbidities were documented preoperatively. Operative data included approach, adjunct procedures, estimated blood loss, intraoperative complications, and duration of surgery. Inpatient length of hospital stay was recorded. All patients were followed up for at least twelve months. Outcomes of symptoms remission, weight gain, recurrence of symptoms, late complications, readmission, and mortality were recorded.

### 2.3. Operative Technique

**Figure 4:**
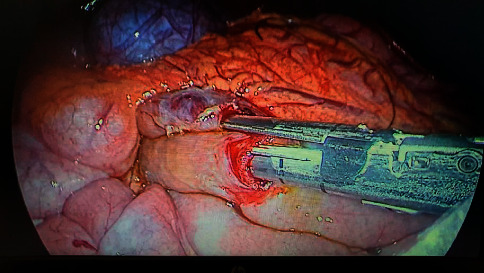
Operative view of the side-to-side stapled duodenojejunostomy.

Patient was positioned in the French split leg position. Surgeon stood between the patient legs and camera holder to the right of the patient. Verres needle was used to establish pneumoperitoneum. An infraumbilical 10 mm camera port was inserted, followed by a 12 mm and a 5 mm ports under vision to the left and right subcostal regions, respectively, to achieve adequate triangulation. Procedure started with retracting the greater omentum and transverse mesocolon cephalad to gain access to the 3^rd^ part of the duodenum. The latter can be identified to the right side of the SMA pulsation. Mobilization of the third and second part of the duodenum was started by division of the overlying visceral peritoneum using a harmonic scalpel. After the duodenum was freely mobilized, a suitable segment of the jejunum about 30 cm from duodenojejunal junction was identified for anastomosis. The standard anastomosis was intracorporeal stapled side-to-side duodenojejunal anastomosis to the proximal third part of the duodenum (Figure 4).

Two stay sutures were placed at the intended sites using 2/0 PDS. Enterotomies to both the limbs were done using a harmonic scalpel. A 45 mm EndoGIA echelon stapler was advanced via the 12 mm port and accurate opposition of bowel loops was checked before stapling. Hemostasis was checked. Common enterotomy was closed using a single layer of continuous 2/0 PDS suture. Nonsuction drain was placed near the anastomosis. The sheath was closed using 2/0 PDS for the 10 mm and 12 mm ports.

Postoperative: patients were allowed free fluids from day one and then build up to normal diet as tolerated. Drain was removed after full diet was established.

## 3. Results

### 3.1. Baseline Demographics

The median age was 23 years (range 17–43). The median BMI was 19 kg/m^2^ (range 15–27 kg/m^2^) (Table 1). The male-to-female ratio was 10 : 1. Duration of symptoms ranged from 12 to 60 months, median 18 months. All patients presented with symptoms of vomiting (*n* *=* 11, 100%), and all except one presented with abdominal pain (*n* *=* 10, 90.9%). One patient had a previous Strong's procedure which had failed to resolve symptoms. The psychiatric preoperative assessment did not reveal any relevant psychiatric condition in any of the patients. Arterial phase CT imaging showed a median aortomesenteric angle of 21° (range 13–28°). All patients had a trial of conservative management for a minimum of 2 months before surgical management was considered.

### 3.2. Intraoperative

Operative time ranged from 125 to 285 minutes and a median operative time of 160 minutes (Table 2). Median recorded blood loss was 70 mls (range 50–160 mls). There were no intraoperative complications reported and no laparoscopic to open conversions.

### 3.3. Postoperative Follow-Up

Median follow up was 16 months (range 4–48 months) (Table 3). Ten out of the eleven patients (*n* *=* 10, 91%) have experienced improvement of their symptoms postoperative, with 8 patients (73%) had complete resolution and no recurrence of symptoms at the latest follow-up appointment. One patient reported occasional vomiting and another had recurrent abdominal pain at follow-up. Only one patient did not have significant symptomatic improvement after 16 months follow-up, despite achieving some weight gain (BMI increase 1.2). All patients gained weight postoperative. Median BMI improved by 2 kg/m^2^ (range 1–9 kg/m^2^). No postoperative complications, readmission, or mortality were recorded.

## 4. Discussion

SMA syndrome is a rare syndrome with limited literature available [2]. SMA syndrome is mainly presented in the form of case reports and case series. These cases are from varying parts of the world, suggesting that the syndrome has a limited correlation to ethnicity. Our case series is, to our knowledge, the largest case series in a cohort of Middle Eastern origin.

The underlying mechanism of the disease is loss of the mesenteric fat pad which results in narrowing of the superior mesenteric angle, thereby, occluding the third part of the duodenum. This sequala of events resulting in SMA syndrome is most often seen in patients with weight loss and low body mass index (BMI) (source). In contrast, our patients presented with a median BMI of 19 kg/m^2^ (range 15–27 kg/m^2^) and seven of our patients had a normal BMI at diagnosis. This is also represented in the literature with several cases of SMA syndrome presenting in patients with a normal BMI [3, 9].

Young and adolescent females are often described as the most common demographic group of patients with SMA syndrome [2]. However, the condition is also seen in males and older age groups (source). Our patient group had a mean age of 27.3 years (range 17–43 years) and a large female majority with a female-to-male ratio of 10 : 1. Two of our patients were over 40 years old (42 and 43 years), which is older than typically described in the literature but much younger than the oldest patient presented at 91 years [4].

In agreement with the available literature, 91% (*n* = 10) of our patients presented with postprandial abdominal pain and vomiting, the most commonly reported symptoms (source). As symptoms are nonspecific, a delayed diagnosis may occur. Delayed diagnosis may have fatal outcomes due to increased risk of aspiration pneumonia with consistent vomiting, gastric rupture, or duodenal perforation and has been reported to be as high as 33% if delayed diagnosis occurs [10, 11].

Ongoing symptoms may also perpetuate further weight loss which may cause further narrowing of the SMA angle. Even though radiological imaging is used to diagnose the syndrome, there is a lack of correlation between severity of symptoms and aortomesenteric angle observed [9]. In healthy individuals, this angle is between 38° and 60° and an angle less than 25° (6°–25°) suggests SMA syndrome [12].

Figure 1 shows a sagittal CT scan of one of our patients with an aortomesenteric angle of 14°. Our case series showed a median aortomesenteric angle 21° (range 13–28°), well below the normal range. Main aim of treatment for SMA syndrome is to relieve the extrinsic obstruction of the third part of the duodenum by the aorta. This is initially attempted through a period of conservative management which focuses on symptomatic relief, correcting the abnormal physiology secondary to weight loss and vomiting, and weight gain and thereby increase of the mesenteric fat pad. Lee et al. defined successful outcome as both weight gain and improvement of symptoms at a 12-month follow-up and report an overall success rate of 71.3% in 80 patients with medical management [3].

The eleven patients in our cohort had at least 12 months of symptoms prior to diagnosis (range 12–60 months) and all failed conservative management. This concurs with reports that conservative management is more likely to be successful with a shorter duration of symptoms prior to diagnosis [13].

Duration of conservative management is not clearly defined. Ganss et al. suggest no more than 3 months of conservative management [14], whereas Sun et al. describe a shorter time interval on the basis that prolonged conservative management may lead to patient deterioration and poor postoperative outcomes. In line with current literature, our patients were considered for an operative approach after a minimum of a two-month trial of conservative management.

Different operative approaches have been described in the literature; Mobilization of the duodenum by division of ligament of Treitz (Strong's procedure) [2], open or laparoscopic gastrojejunostomy, or duodenojejunostomy [2, 15], [16]. Strong's procedure has the benefit of not requiring an anastomosis and is associated with a short postoperative recovery; however, it is not always possible due to adhesions or short vascular connections between the inferior pancreaticoduodenal artery and duodenum [7].

Gastrojejunostomy can provide symptomatic relief of a distended stomach, although it does not relieve the duodenal obstruction [17]. It can be considered if other procedures prove difficult such as cases where the duodenum is significantly distended increasing the risk of anastomotic leak [15, 18].

Duodenojejunostomy is technically more challenging but is more physiological than gastrojejunostomy and does not carry the risk of bile reflux often seen in the latter procedure [16]. With advancement in laparoscopic surgery, a minimally invasive approach has become more favourable, but there are only limited numbers of case reports and case series available in the literature.

A PubMed search using the search word laparoscopic duodenojejunostomy found 109 articles with less than 180 cases total describing the minimal invasive treatment approach in SMA syndrome [19], [20], [21], [22].

## 5. Conclusion

SMA syndrome is a significant condition with severe adverse outcomes if not diagnosed and correctly managed. We believe that laparoscopic duodenojejunostomy is a better surgical option compared with other procedures described in the literature and should be offered when symptoms are not resolved by conservative management. Laparoscopic duodenojejunostomy is a safe procedure with low morbidity. We show with our case series a good clinical outcome after laparoscopic duodenojejunostomy, both symptomatic relief/remission and sustainable weight gain.

Data collection was done under local ethical approval. The data were retrospectively collected, and obtaining patient's consent was not possible for the scope of this case series.

## Figures and Tables

**Table 1:** Gender, age, presenting symptoms, BMI, and aortomesenteric angle degree in the corresponding CT.

	Age	Sex	Symptoms	Duration of symptoms (months)	BMI(kg/m^2^)	A-M angle (degrees)
	Median = 23	Male-to-female ratio (10:1)		Median = 18	Median = 19	Median = 21
Patient 1	42	Female	Abdominal pain and vomiting	12	20.	—
Patient 2	37	Female	Abdominal pain and vomiting	24	23	13
Patient 3	24	Female	Persistent vomiting after release of ligament of Treitz	24	20	23
Patient 4	18	Female	Abdominal pain and vomiting	36	17	19
Patient 5	43	Female	Abdominal pain and vomiting	18	18	27
Patient 6	35	Female	Abdominal pain and vomiting	24	19	19
Patient 7	21	Female	Abdominal pain and vomiting	12	27	19
Patient 8	23	Female	Abdominal pain and vomiting	12	16	22
Patient 9	17	Female	Abdominal pain and vomiting	12	19	28
Patient 10	18	Female	Abdominal pain and vomiting	12	15	25
Patient 11	23	Male	Abdominal pain and vomiting	60	19	20

**Table 2:** Operative duration and blood loss.

	Duration of the operation (minutes)	Operative blood loss (mls)
	Median = 160	Median = 70
patient 1	—	—
Patient 2	125	90
Patient 3	265	130
Patient 4	285	160
Patient 5	155	80
Patient 6	185	50
Patient 7	165	50
Patient 8	135	70
Patient 9	135	60
Patient 10	149	70
Patient 11	168	50

**Table 3:** Symptoms and BMI improvement postoperative.

	Follow-up duration (months) Median = 16	Symptoms outcome	BMI at follow up (kg/m^2^) Median = 21	BMI difference at follow-up (kg/m^2^) Median = 2
Patient 1	16	Persistent abdominal pain	22	1.2
Patient 2	48	No recurrence	32	9
Patient 3	18	Recurrent abdominal pain	22	2
Patient 4	6	Vomiting occasionally on monthly basis after strenuous effort	18	1
Patient 5	18	No recurrence	—	—
Patient 6	45	No recurrence	26	7
Patient 7	4	No recurrence	—	—
Patient 8	6	No recurrence	19	3
Patient 9	12	No recurrence	21	2
Patient 10	24	No recurrence	21	6
Patient 11	9	No recurrence	20	1

## Data Availability

The data used to support this study are included within the article.
